# Development of Energy Efficient Clustering Protocol in Wireless Sensor Network Using Neuro-Fuzzy Approach

**DOI:** 10.1155/2016/5063261

**Published:** 2016-01-03

**Authors:** E. Golden Julie, S. Tamil Selvi

**Affiliations:** ^1^Department of Computer Science and Engineering, Regional Office, Anna University, Tirunelveli, Tamil Nadu 627 007, India; ^2^Department of Electronics and Communication Engineering, National Engineering College, Kovilpatti, Tamil Nadu 628 503, India

## Abstract

Wireless sensor networks (WSNs) consist of sensor nodes with limited processing capability and limited nonrechargeable battery power. Energy consumption in WSN is a significant issue in networks for improving network lifetime. It is essential to develop an energy aware clustering protocol in WSN to reduce energy consumption for increasing network lifetime. In this paper, a neuro-fuzzy energy aware clustering scheme (NFEACS) is proposed to form optimum and energy aware clusters. NFEACS consists of two parts: fuzzy subsystem and neural network system that achieved energy efficiency in forming clusters and cluster heads in WSN. NFEACS used neural network that provides effective training set related to energy and received signal strength of all nodes to estimate the expected energy for tentative cluster heads. Sensor nodes with higher energy are trained with center location of base station to select energy aware cluster heads. Fuzzy rule is used in fuzzy logic part that inputs to form clusters. NFEACS is designed for WSN handling mobility of node. The proposed scheme NFEACS is compared with related clustering schemes, cluster-head election mechanism using fuzzy logic, and energy aware fuzzy unequal clustering. The experiment results show that NFEACS performs better than the other related schemes.

## 1. Introduction

Wireless sensor networks (WSN) consist of number of sensor nodes with low energy and limited processing capability. WSN is able to sense the physical environment and report about environment data to base station. In critical applications, sensors are randomly deployed in particular region to monitor the environments [1]. Network topology and energy consumption are important issues in WSN for improving network performance in critical applications. Existing clustering schemes are not efficient in terms of energy efficiency. Clustering techniques are playing very important role to maintain the network topology effectively that partition the group of nodes. It maintains the network topology effectively. Artificial neural network (ANN) [2], energy efficient hierarchical unequal clustering [3], continuous time recurrent neural network [4], probabilistic neural network [5], neuro-fuzzy approach [6], and intrusion detection systems based on artificial intelligence technique [7] are classified effectively in WSN. Energy consumption is a great issue in cluster based routing architecture in WSN. It is necessary to develop an energy efficient clustering protocol in WSN for increasing network lifetime.

It is essential to consider energy efficiency for developing cluster based routing schemes in WSN. Many clustering techniques [8] are surveyed in the related works. Energy aware unequal clustering fuzzy (EAUCF) scheme [9] used fuzzy logic to select the cluster heads that considered the residual energy and the distance to the base station of the sensor nodes. Energy efficient cluster formation (EECF) scheme [10] is followed by a three-way message exchange between each sensor and its neighbors. Residual energy and node are considered to select the cluster heads (CHs). The related schemes EAUCF and EECF were not considered a mobility factor to form clusters that do not provide better solution. Balan et al. [11] proposed a system to detect the malicious behavior of node by intrusion detection system with fuzzy logic technique and identified the attacks such as black hole attack and gray-hole attack. This system also prevents the attacks by using efficient node blocking mechanism.

In this paper, a neuro-fuzzy energy aware clustering scheme (NFEACS) is proposed to form clusters and cluster heads. NFEACS is integrated with neural network system that provides expected energy for tentative cluster heads. Then, fuzzy set approach is used to form clusters and cluster heads that make energy efficiency. NFEACS considered the mobility factor of nodes in WSN. In order to make energy efficiency, the proposed approach uses the mobility factor and residual energy of the sensor nodes to form clusters.

This paper is organized as follows. In Section 2, we present related work related to energy aware clustering schemes. In Section 3, we present proposed NFEACS. In Section 4, we present the simulation results and discussion. Finally, Section 5 presents conclusion and future directions.

## 2. Related Works

Energy efficient clustering schemes for WSN are widely discussed in the literature. The objective of clustering is the effective topology management and intracluster routing. A fuzzy based clustering protocol is proposed [12] for enhancing energy-efficiency in wireless sensor networks that combines both clustering method and fuzzy logic. Mobility sensor nodes are not considered in scenario. Lee and Cheng [13] proposed a fuzzy-logic-based clustering approach for WSN for energy predication. Many energy aware clustering schemes such as multiobjective fuzzy clustering [14], genetic algorithm [15], fuzzy logic for combining particle swarm optimization and genetic algorithms [16], energy efficient routing protocol with data aggregation [17], and interval type-2 fuzzy logic systems [18] are proposed for addressing energy consumption issues. Nekooei and Manzuri-Shalmani [19] proposed soft computing methods for increasing the network lifetime in WSN.

Low energy adaptive clustering hierarchy (LEACH) [3] was mainly focused on energy metric to form clusters and cluster heads. It also considered load balancing mechanism when cluster heads are elected using rotation basics. Cheng et al. [20] proposed an algorithm that adapts operators of genetic approach with elitism strategy into the iteration in tracking weights. Power efficient gathering in sensor information systems (PEGASIS) [17] was an enhancement of LEACH that forms chains of sensor nodes for transmitting and receiving nodes. It was not suitable for large networks and it wastes much energy to form clusters and cluster heads. Hybrid energy efficient distributed clustering (HEED) [21] used a combined metrics such as residual energy and intracluster communication cost for selecting clusters and cluster heads. Works in [22, 23] proposed fuzzy clustering schemes that use unsupervised learning for remote sensing images when the changes are detected. It was not handling group redundancy in WSN. Sert et al. [14] presented multiobjective fuzzy clustering algorithm to solve hotspots issues in WSN. It consumes more energy in the presence of mobile sensor. Alrajeh et al. [24] presented an artificial neural network based detection of energy exhaustion attacks in WSN that is capable of energy harvesting. They are not considered for mobility of sensors.

## 3. Proposed Neuro-Fuzzy Energy Aware Clustering Scheme

### 3.1. Sensor Network Model

The assumptions of that network model are listed:

- Mobile sensor nodes are randomly deployed with the same energy level.
- Each sensor node had moved frequently in region.
- Distances between nodes were computed based on the received signal strength.
- The base station needs to be located at the center of the region.

The proposed scheme used Energy aware unequal clustering fuzzy (EAUCF) scheme [9] with some modification. Neural network system is integrated in EAUCF for training network related to energy and received signal strength of all nodes. The radio model [9] is used in simulation for consuming energy. The energy consumed during transmission and reception for a *k*-bit message to a distance *d* between transmitter and receiver node is given by

(1) ETxk,d=Eelec∗k+εamp∗k∗d,ERxk=Eelec∗k.

### 3.2. Neural Network System

The supervised learning approach is used to train the network. Single layer perceptron learning rule is used to train the network [2]:

(2) $$\begin{array}{c} W_{i}\left(\;t-1\;\right)=W_{i}\left(\;t\;\right)+\mu \times \left(\;{\text{O}}_{T}-{\text{O}}_{A}\;\right)\times L, \end{array}$$

where *W*_*i*_ is the weight factor of *i*th cell, *i* is the number of input cells, *μ* is the learning rate, *L* is the input of that cell, O_*A*_ is the output of the network, and O_*T*_ is the desired output.

The adjustment of weight is given by

(3) $$\begin{array}{c} W_{\text{new}}=W_{\text{old}}+N\times \mu \times I_{p}\times \left(\;{\text{O}}_{T}-{\text{O}}_{A}\;\right), \end{array}$$

where

(4) $$\begin{array}{c} \Delta W=N\times \mu \times I\times \left(\;{\text{O}}_{T}-{\text{O}}_{A}\;\right). \end{array}$$

*N* is the active neuron, and *I*_*p*_ is the input from the previous layer to that active neuron.

Output rate in each layer is expressed as

(5) $$\begin{array}{c} f_{1}\left(\;I_{1}\times W_{1}\;\right)\\ f_{2}\left(\;I_{2}\times W_{2}\;\right)\\ f_{3}\left(\;I_{3}\times W_{3}\;\right). \end{array}$$

The system has three inputs. The inputs such as the residual energy and received signal strength of sensor nodes are trained by neural network. Input to the system represents energy consumption in network as a continuous variable. Best quality link between node is measured by radio signal strength that is found by

(6) $$\begin{array}{c} L_{q}=\frac{S_{p}}{S_{\max}^{}}, \end{array}$$

where *S*_*p*_ is the signal strength and *S*_max⁡_ is the maximum strength available.

**Figure 1:**
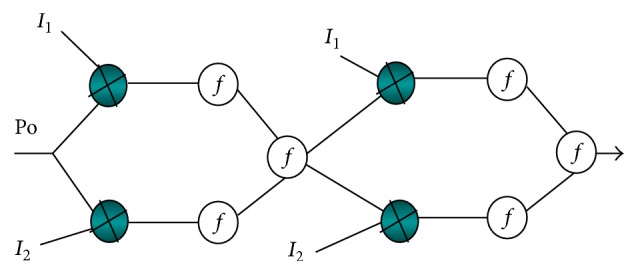
One cell of network layer.

Finally, the efficient link quality and also maximum energy sets get trained to select efficient nodes for tentative cluster heads that represent the reliable transmission between source and destination. This input is connected to *n* first inputs of neural networks. The second input is location of base station that is discrete valued and the last input is feedback outputs. Three inputs make different scenarios in different conditions (Figure 1).

The two upper neurons have fixed weights with linear function. The lower two neurons have trainable weights that were used in sigmoid function of neural network. Neurons can map a set of inputs to a desired output. The sensor network was trained with a scenario-like base station that is located at the centre of the environment. Single layer perceptron is used to train the network to get desired output.  Algorithm 1 shows the perceptron learning rule and weight change rule. Network trained and maps outputs with previous learning procedure. The proposed neural network gives energy efficient cluster heads and reliability that is important in a WSN. Result in neural network is the best energy efficiency scenarios for WSN.

### 3.3. Fuzzy Logic System

The proposed scheme used energy aware unequal clustering fuzzy (EAUCF) algorithm [10] which is integrated with mobility factor. Sensors are randomly deployed in an environment. The sensors are allowed to move freely when they are required. The proposed system chooses a location for a base station at centre of the environment. NFEACS used fuzzy logic approach to select cluster heads.  Table 1 shows the fuzzy if-then mapping rule for cluster head chance in NFEACS. NFEACS is on a neural network that provides desired threshold to elect the cluster heads. In this, we considered residual energy and mobility factor weight factors to form efficient cluster heads.

The clustering algorithm is shown in Algorithm 2. It selects a cluster head with low mobility factor and high residual energy.

NFEACS uses residual energy and mobility factor to select the cluster heads. It is derived from fuzzy if-then mapping rule. Fuzzy system used two fuzzy input variables such as mobility factor and residual energy. The fuzzy set linguistic variables such as high, medium, and low are considered for mobility factor. A trapezoidal membership function is chosen for low and high variables. The triangular membership function is selected for medium variable. The linguistic variables like high, medium, and low are selected for residual energy.

**Figure 2:**
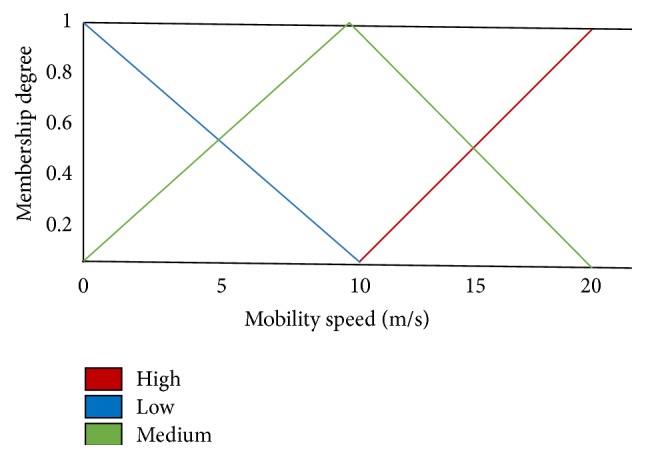
Fuzzy set for fuzzy input variable mobility speed.

**Figure 3:**
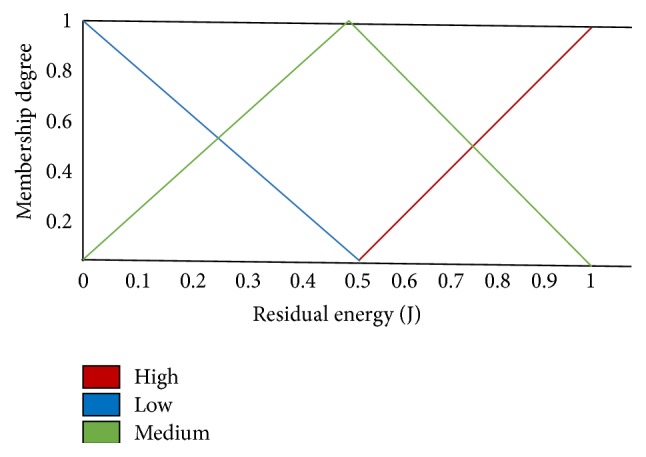
Fuzzy set for fuzzy input variable residual energy.

**Figure 4:**
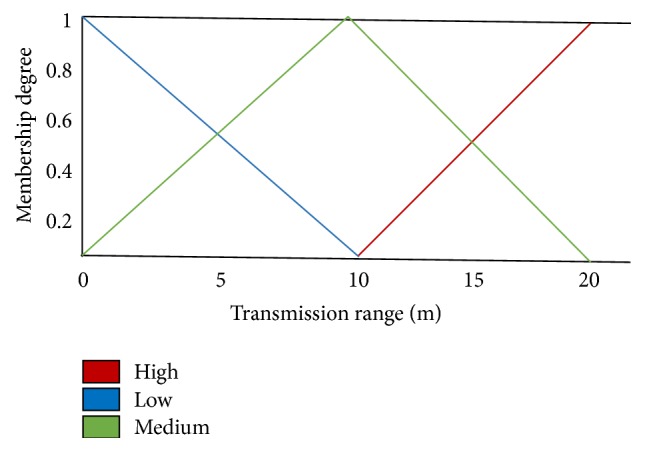
Fuzzy set for fuzzy input variable transmission range.

Figure 2 shows the fuzzy set input variable for mobility factors that are high, medium, and low. Triangular membership function is chosen for medium linguistic variable. A trapezoidal membership function is chosen for low and high variables.  Figure 3 illustrates that the residual energy is an input variable for fuzzy set. Trapezoidal membership function is applied for low and high linguistic variables and triangular membership function is chosen for medium linguistic variable.  Figure 4 illustrates that the transmission range is also an input variable for fuzzy set. High variable represents a node with maximum transmission range of about more than 10 m selected as cluster head. Membership functions are tested to get the most excellent functions for input variables that give good result.

## 4. Simulation Results

The proposed scheme NFEACS used neuro-fuzzy system for enhancing the energy efficiency in forming effective clusters. Sensor nodes are varied from 50 to 200 sensor and they were randomly deployed in a 500 m × 500 m simulation area. The transmission range was 20 m. The neuro-fuzzy energy aware clustering scheme (NFEACS) was evaluated and compared with related schemes such as energy aware unequal clustering fuzzy (EAUCF) scheme and energy efficient cluster formation (EECF) scheme. The base station is located at the center of the region of interest. All sensor nodes are initially assigned with 1 J. The NFEACS integrate neural network system with EAUCF scheme instead of probabilistic model for selecting tentative cluster head. Neuro-fuzzy approach was used in NFEACS to get energy aware clusters and cluster heads. In our simulations, we used the same parameter values as [2] such as *ε*_amp_ = 100 pJ/bit/m^2^,  *E*_elec_ = 50 nJ/bit, and aggregation ratio 10% and mobility factor is also considered that is not considered in EAUCF.

### 4.1. Cluster Overhead

**Figure 5:**
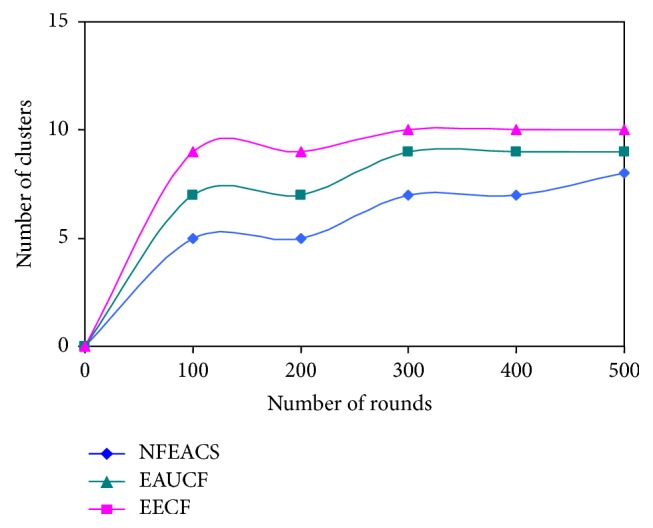
Number of clusters versus number of rounds.

Figure 5 shows number of clusters with respect to the number of rounds for proposed scheme and related schemes. EAUCF and EECF generate the higher number of clusters at each round compared to NFEACS. NFEACS generates the minimum clusters in each round. As the number of rounds increases, NFEACS forms minimum clusters and cluster heads when the number of rounds is increased. NFEACS used training set that provides accurate weight value with respect to residual energy and mobility factor for selecting cluster heads, whereas the EAUCF used only fuzzy approach for selecting clusters and cluster heads that provide higher number of clusters than NFEACS. It causes overhead in packet forwarding in the environment.

### 4.2. Network Lifetime

**Figure 6:**
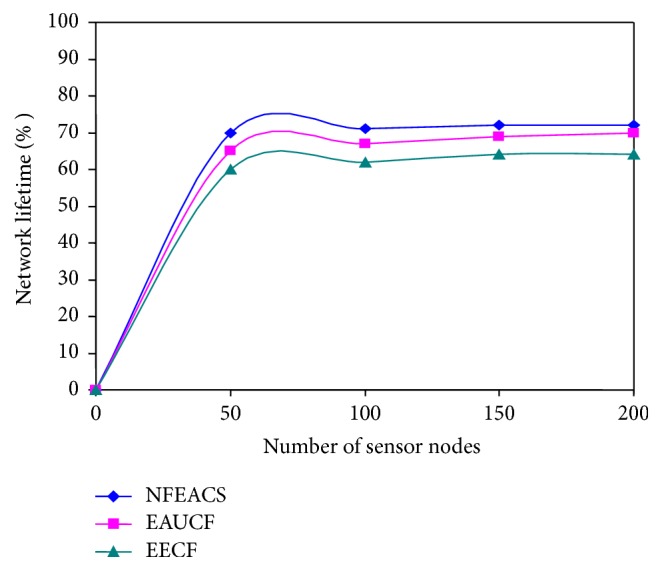
Network lifetime with respect to number of sensors.

Figure 6 shows network lifetime with respect to varying number of sensors. It is used to assess the efficiency of the NFEACS in terms of network lifetime. In our simulation, numbers of sensor nodes are varied such as 50, 100, 150, and 200. Performance of NFEACS is evaluated with efficiency of network lifetime with respect to ratio of number of CHs among total number of sensors nodes in the network.  Figure 4 notices that NFEACS is very consistent with respect to network lifetime because it used neuro-fuzzy approach to elect the optimal cluster heads compared to other related schemes. Network lifetime is increased when the nodes are increased. NFEACS save 20% of network lifetime compared to related schemes.

### 4.3. End-to-End Delay

**Figure 7:**
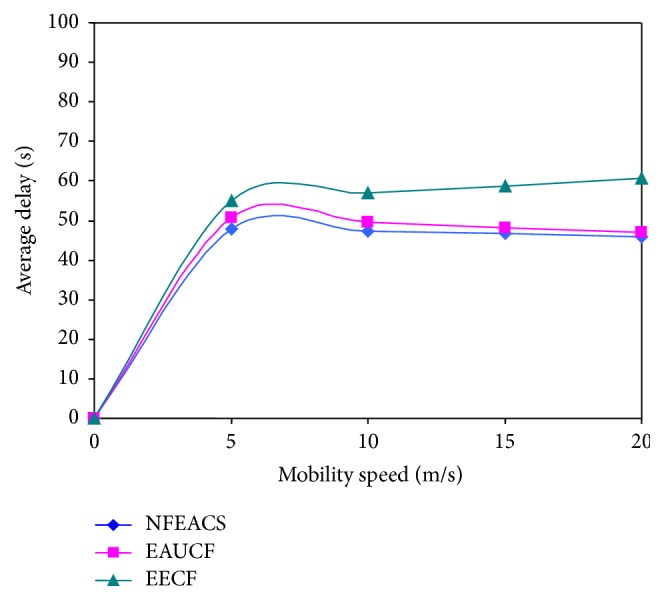
Mobility speed versus delay.

In our simulation, the proposed scheme assumes sensor nodes are mobiles that traverse the network.  Figure 7 shows that mobile speed of sensor nodes is varied from 0 to 20 m/s. NFEACS has minimum delay for forwarding packets from destination to base station compared with related schemes because it used neural network approach that provides cluster head reference to select optimal cluster heads in all clusters. NFEACS achieved minimum energy consumption because of low mobility factor nodes with high residual energy that is elected as cluster heads. It consumes minimum energy that increases network lifetime. The NFEACS has minimum delay even when the sensor nodes are mobile in network. NFEACS also used fuzzy approach to select the low mobility node and neural network to form efficient clusters and cluster heads.

### 4.4. Packet Drop Rate

**Figure 8:**
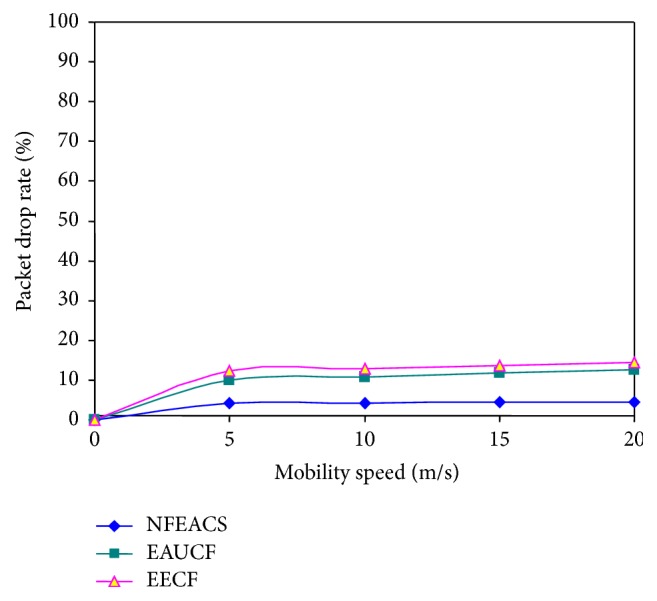
Mobility speed versus packet drop rate.

Figure 8 shows that the NFEACS has minimum packet drop rate compared with related schemes because the proposed scheme used fuzzy approach to select node with low mobility for selecting cluster heads. The related scheme has more packet drop rate when it considers mobility factor of the sensor nodes. Figure 6 notices that the NFEACS has minimum packet drop rate than related scheme when sensor nodes are mobile in network. NFEACS achieved 37% energy efficiency compared to related schemes.

### 4.5. Number of Alive Nodes

**Figure 9:**
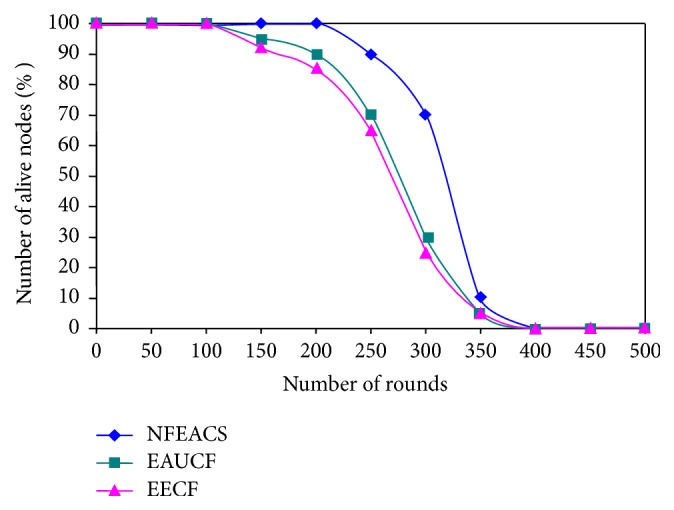
Number of alive nodes in each round.

Figure 9 shows number of alive sensor nodes in each round. It clearly noticed that NFEACS outperforms related schemes because it had alive nodes up to 450 rounds.  The related schemes all the sensor nodes are death in 400 rounds that degrade the network lifetime. NFEACS used neuro-fuzzy system that considers three types of metrics such as transmission range, residual energy, and mobility factor to form clusters and cluster heads. The related schemes did not consider the mobility factor to form clusters.

### 4.6. Signal Strength Ratio

**Figure 10:**
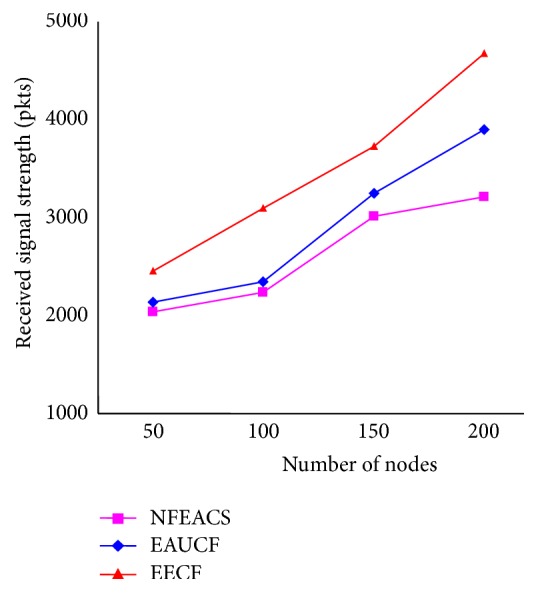
Signal strength ratio.

Figure 10 shows the signal strength ratio (SSR) by varying the nodes in the WSN. It shows that the proposed schemes maintained more SSI than related schemes. The SSI of NFEACS had minimum routing packets for transmission compared to related schemes. In related schemes, more routing packets are generated for making transmission that causes routing overhead. It shows that the NFEACS provides more link quality between nodes for involving cluster formation and cluster head selection. The related schemes have less SSI because it was not considered as link quality to clusters. Normally the value of SSI gets decreased in the proposed scheme since it sends more numbers of data in a time when being compared to the related schemes.

Table 2 shows the total remaining three schemes. The proposed scheme NFEACS performs better than EAUCF and EECF with respect to mobility and energy metrics. EECF consumes minimum energy and has higher remaining energy about 48.54 J compared to the other two schemes like NFEACS and EAUCF. The proposed scheme NFEACS is more efficient than the other two related schemes about 37% if neuro-fuzzy approach is considered for selecting node with low mobility. EAUCF provides efficient clustering approach than EECF with about 28.5% of energy efficiency because it is considered the fuzzy scheme. The EECF performance is the poorest, because it does not use fuzzy approach during clustering.

## 5. Conclusion

We proposed a novel scheme named NFEACS that considered neuro-fuzzy approach to form clusters and select energy aware cluster heads. NFEACS analyzed the network lifetime of WSN and the effectiveness of clustering and energy of network is investigated. NFEACS could significantly select energy aware clusters because it used fuzzy system for selecting sensor with low mobility as cluster heads. The feature of neural network can be used to provide cluster head reference that yields optimum results in terms of energy consumption in WSN. We concluded that NFEACS has an increase of 37% in average of energy saving compared to related schemes. Furthermore, the proposed results analyse the energy efficiency of the proposed scheme using the parameters such as energy consumption, packet drop rate, and network lifetime of WSN. A possible future research direction can add weighted metrics like node degree and received signal strength would be used in neural network training set.

## Figures and Tables

**Algorithm 1:**
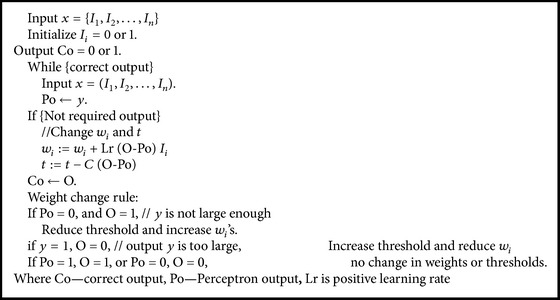
Perceptron learning rule.

**Algorithm 2:**
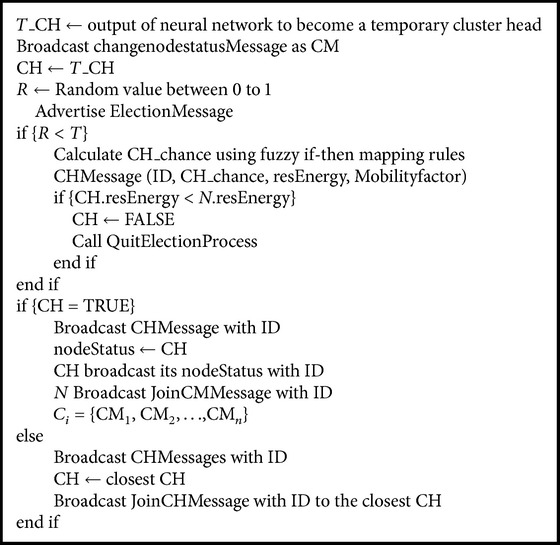
Clustering algorithm of NFEACS protocol.

**Table 1:** Fuzzy if-then mapping rule for cluster head chance.

Mobility factor (speed/sec)	Transmission range (m)	Residual energy (J)	Cluster head chance
Low	High	Low	Medium
Medium	Medium	High	High
Medium	Low	High	Medium
Medium	High	High	Low
Medium	Medium	Medium	Low
Medium	Low	Medium	Low
Medium	High	Medium	High
Medium	Medium	Low	High
Medium	Low	Low	Medium
Medium	High	Low	High
High	Medium	High	High
High	Low	Medium	Medium
High	High	Medium	Medium
High	High	Medium	High
High	Medium	Low	Medium
High	Low	Low	Low

**Table 2:** Total remaining energy for three schemes.

Algorithm	Number of alive nodes	Total remaining energy (J)
NFEACS	110	48.54
EAUCF	100	41.19
EECF	80	39.96
